# Formation of Highly Ordered Platinum Nanowire Arrays on Silicon via Laser-Induced Self-Organization

**DOI:** 10.3390/nano9071031

**Published:** 2019-07-18

**Authors:** Michael Dasbach, Hendrik M. Reinhardt, Norbert A. Hampp

**Affiliations:** 1 Department of Chemistry, Philipps-University of Marburg, Hans-Meerwein-Str. 4, 35032 Marburg, Germany; 2 Material Science Center, 35032 Marburg, Germany

**Keywords:** laser-induced periodic surface structures, platinum nanowires, self-organization

## Abstract

Laser-induced periodic surface structures (LIPSS) provide an elegant solution for the generation of highly ordered periodic patterns on the surface of solids. In this study, LIPSS are utilized for the formation of periodic platinum nanowire arrays. In a process based on laser-stimulated self-organization, platinum thin films, sputter-deposited onto silicon, are transformed into nanowire arrays with an average periodicity of 538 nm. The width of the platinum nanowires is adjustable in a range from 20 nm to 250 nm by simply adjusting the thickness of the initial platinum thin films in a range from 0.3 nm to 4.3 nm. With increasing width, platinum nanowires show a rising tendency to sink into the surface of the silicon wafer, thus indicating alloying between platinum and silicon upon LIPSS-formation by a nanosecond-pulsed laser. The Pt/silicon wires may be etched away, leaving a complementary nanostructure in the silicon surface.

## 1. Introduction 

Miniaturization is a must in modern technology, hence viable processes for the manufacture of nanostructures gain ever-increasing importance. Large surface areas, abundant surface states and quantum behavior make nanomaterials indispensable in a wide range of applications [1]. Examples include photonic applications where nanowires are utilized to improve light emission or rather light harvesting, as well as wave propagation, thus improving the performance of photodetectors, waveguides, LEDs, microcavity lasers, optical converters, and solar cells [2,3,4]. Highly ordered nanowires have great potential as miniaturized conducting tracks and field effect transistors, thus providing a promising basis for next generation chemical, biochemical and gas sensors [5,6,7]. Due to their one-dimensional structure, nanowires resemble highly effective thermoelectric pathways, which facilitate the efficient conversion of temperature gradients into electrical energy [8,9]. Nanowires are used as high performance anode materials in rechargeable lithium batteries as they improve the storage density of lithium, hence increasing the capacity of batteries. High lithium densities typically cause mechanical degradation of batteries due to volumetric strain, which can be compensated by the addition of nanowires [10]. Highly ordered nanowires, or rather nanopatterned surfaces, required in the mentioned fields of applications, are commonly manufactured by lithographic processes. Lithography is, however, a cost-intensive and time-consuming multistep process [11,12] almost exclusively applicable to mass products like classical semiconductors [13]. For these reasons, there is great demand for patterning techniques overcoming the limitations of lithography. Laser induced periodic surface structures (LIPSS) offer an elegant alternative since this method of pattern formation is of comparably low-cost, highly flexible and is a single step process [14]. First discovered by Birnbaum in the mid-1960s [15], the process of LIPSS-generation today is applicable to a wide variety of materials, such as metals, alloys, semiconductors, dielectrics and polymers [16,17,18,19,20,21,22]. While the mechanism of LIPSS-formation is still not fully understood in every detail [14,20,23,24,25,26], the potential of this self-organization phenomenon is already applicable for practical applications. Most theories of nanopattern formation suggest an interference between the incident/reflected laser beam and the scattered/diffracted light near the sample surface, resulting in a periodic energy pattern [23]. For LIPSS generated at a metal-dielectric interface, the excitation of surface plasmon polaritons SPP is considered [24]. Due to the resulting periodic energy profile, the material self-organizes into periodic nanowires. The driving force for this process is typically considered thermally, providing wires in the relative cold regions of the surface formed due to the periodic energy entry of the laser wave; however, a photonic mechanism in form of an optical tweezer behavior is also possible, in which the material gets gathered in the high energy regions [14,27]. Since both forces are present when considering a formation via a nanosecond laser, the exact mechanism of LIPSS generation of the theoretical modelling is not finalized. Depending on specific requirements, a choice of two patterning regimes is available: Low spatial frequency LIPSS (LSFL) and high spatial frequency LIPSS (HSFL). The generation of LSFL is commonly performed with nanosecond-pulsed lasers whereas femtosecond pulsed lasers generate HSFL [28]. Besides the type of laser, the main distinctive feature between these regimes is the achievable pattern periodicity, which is typically close to the laser wavelength in the case of LSFL, but may reach even 20 nm when HSFL is employed. The periodicity *Λ* of patterns generated in the LSFL regime can be well predicted using the equation *Λ* = *λ* ∙ [(*n* ∙ (1 − sin*θ*)]^−1^, where *λ* is the laser wavelength, *n* the refraction index of the irradiated material and *θ* the angle of laser beam incidence [16,29]. 

In this study, we demonstrate the formation of periodic platinum nanowire arrays on silicon. All patterns are generated in the LSFL regime via nanosecond-pulsed laser stimulation of platinum thin films sputter-deposited on silicon wafers. The correlation between the thickness of platinum thin films and the width of the resulting platinum nanowires is presented. Nanowire widths in the range from 20 nm to 250 nm are generated by adjusting the thickness of sputter-coated platinum in the range from 0.3 nm to 4.3 nm. Cross-sectional analyses reveal that platinum nanowires are embedded into the surface of the silicon substrate, thus indicating transient temperatures sufficiently high for alloying platinum and silicon to arise upon LSFL formation. TEM analysis indicates the amorphization of the silicon wafer surface in a periodic manner between the platinum lines, corresponding well to the thermal LIPSS generation theory.

## 2. Materials and Methods 

Mechano-chemically polished boron-doped silicon wafers (P-type, 1-0-0, 650 ± 25 μm thickness, 2.1 Ω cm^−2^ Siltronic AG, Munich, Germany) were coated with platinum films of different thicknesses using a sputter coating system (ALTO 2500, Gatan, Milton, UK). The thickness of each film was measured by single wavelength ellipsometry. A frequency doubled nanosecond-pulsed Nd:YVO_4_-laser, emitting laser pulses of 8 ns pulse width at a wavelength of 532 nm, was used for LIPSS-formation (Explorer XP 532-5, Newport, Irvine, USA). The laser beam was scanned over platinum-coated silicon wafers with a line spacing of 3 μm and a scan speed of 10 mm s^−1^ using a galvanometer scan head (SCANgine 14-532, Scanlab, Puchheim, Germany) equipped with an F-Theta lens (Rodenstock, f = 163 mm, Munich, Germany), which focused the laser beam to an effective spot diameter of 30 μm (1/e^2^). All samples were modified using a laser fluence per pulse of φ = 2.74 J cm^−2^, a pulse repetition rate of *f* = 50 kHz and an average rate of energy transfer per unit area of Φ_area_ = 447 MJ cm^−2^. The peak power of the laser is P_Peak_ = 2425 W. A scheme of the used laser setting is given in the Supplementary Materials. For etching studies, the samples were treated with freshly prepared aqua regia for 35 s, followed by hydrogen fluoride (1 M) for 18 min at room temperature. Samples were analyzed on a field emission scanning electron microscope (SEM, JSM-7500F, Jeol, Akishima, Japan) equipped with secondary and backscattered electron detectors and a scanning transmission electron microscope (STEM, JEM 2200FS, Jeol, Akishima, Japan). Focused Ion Beam (FIB) slices were prepared on a Zeiss Crossbeam 550 (Zeiss, Oberkochen, Germany).

## 3. Results and Discussion

**Figure 1:**
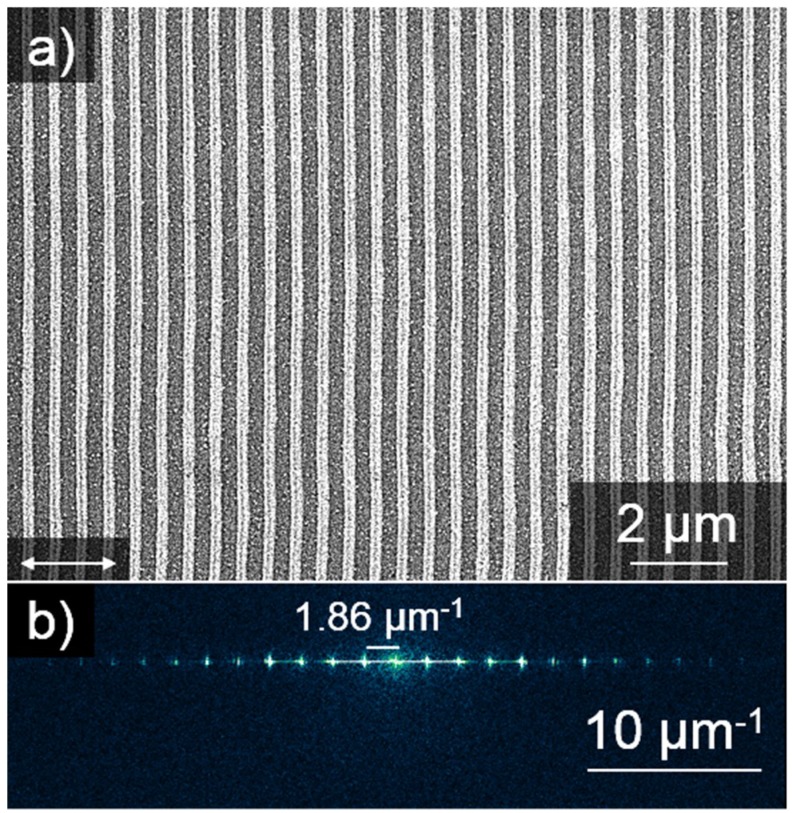
Pt-nanowire array on silicon. (**a**) SEM image of laser-induced periodic surface structures (LIPSS) generated from a 2.2 nm thin layer of platinum on silicon. Pt-nanowires are oriented orthogonally with respect to the laser polarization (white double arrow). (**b**) A 538 nm periodicity of the Pt-Nanowires is obtained from the Fast Fourier Transformation (FFT) of the SEM image above.

A typical result of LIPSS formation from a 2.2 nm platinum thin film is shown in Figure 1. Pattern analysis via Fast Fourier Transformation (FFT) returns an average nanowire periodicity of 1.86 µm^−1^ corresponding to 538 nm, which is close to the laser wavelength, thus matching theoretical predictions for LIPSS formation in the LSFL regime [16,29]. The orientation of the Pt-nanowires is perpendicular to the laser polarization. All nanowire arrays investigated in this study feature the same periodicity and orientation. Minor wiggles in nanowire development may be caused due to minor imperfections of the Pt thin layer, mainly caused by the laser illumination itself. However, the very straight-lined nanowires presented in this work may be produced in constant quality even on cm-sized areas.

**Figure 2:**
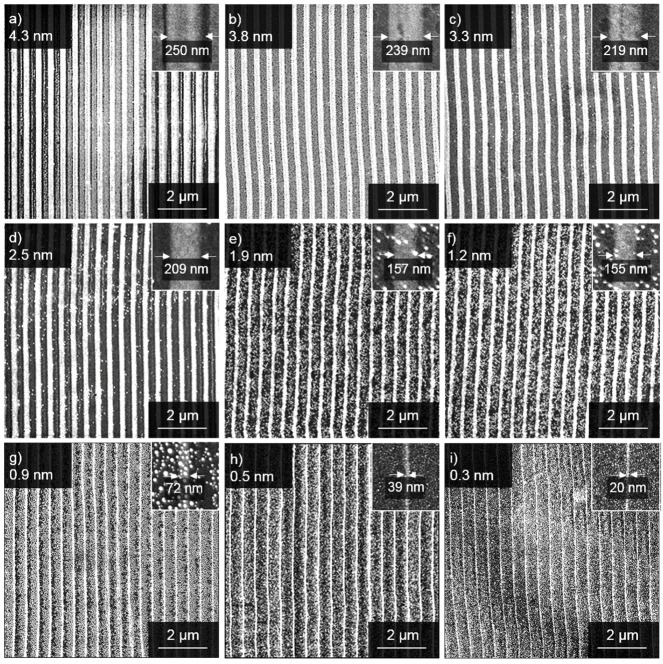
SEM images of Pt-nanowire arrays obtained by the LIPSS process from silicon supported Pt-films of varying thicknesses (indicated top left in each image). Detailed views as well as the measured line width are given in the top right corner inserts. The upper left corner of each image indicates the initial Pt-layer thickness of (**a**) 4.3 nm, (**b**) 3.8 nm, (**c**) 3.3 nm, (**d**) 2.5 nm, (**e**) 1.9 nm, (**f**) 1.2 nm, (**g**) 0.9 nm, (**h**) 0.5 nm, (**i**) 0.3 nm.

**Figure 3:**
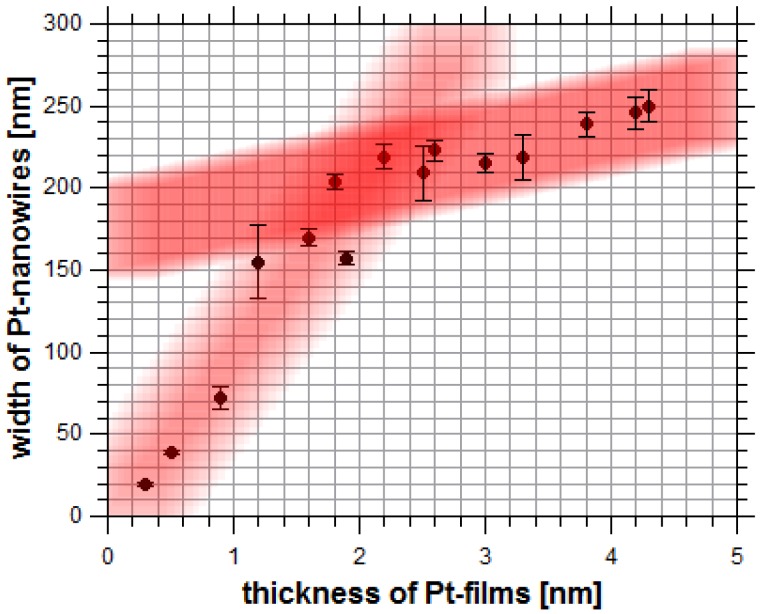
Dependence of the Pt-nanowire widths on the initial Pt-film thicknesses. The cross-section of both red lines indicates a change in the formation mechanism of LIPSS.

**Figure 4:**
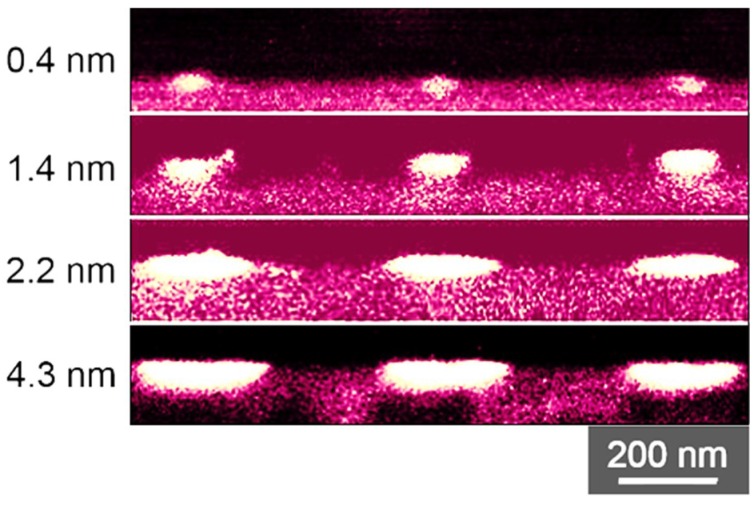
Backscattered electron images of Pt-wire cross-sections in relation to the initial Pt-films thicknesses.

In order to investigate the influence of the film thickness on the widths of the formed Pt-nanowires, a set of 15 samples was sputter-coated with platinum films ranging from 0.3 nm to 4.3 nm thickness, measured by single wavelength ellipsometry. In Figure 2, a representative set of Pt-nanowire arrays is shown, which were obtained by the LIPSS process. An influence of the initial film thickness on the widths of the resulting Pt-nanowires is obvious. Nanowires formed from 4.3 nm Pt-films feature a width of 250 nm, while nanowires formed from thinner Pt-films show smaller widths down to a minimum of 20 nm, obtained from a Pt thin film of 0.3 nm thickness. Minor fluctuations in the width of the Pt-nanowires were taken into account by averaging the widths over ten individual nanowires. The relation between Pt-film thickness and the resulting nanowire width is plotted in Figure 3. For low film thicknesses from 0.3 nm to 2.2 nm, a direct relation to the nanowire width obtained is found. For higher Pt-film thicknesses, only a relatively small further increase in the widths of the resulting nanowires is observed, thus indicating a self-limiting structure broadening. Obviously, LIPSS-stimulated nanowire formation in the system Pt/Si is a rather complex process. The self-limitation of wire widths is seen in the cross-sectional analyses of the formed Pt-nanowires (Figure 4). With increasing amounts of Pt material available, i.e., higher Pt-film thicknesses, the nanowires appear halfway sunken into the surface, thus indicating alloy formation between platinum and silicon. For thinner Pt-layers, the obtained nanowires tend to occur mainly on top of the wafer surface in a more or less spherical shape, which probably is caused by a surface tension-driven self-organization phenomenon. For increasing amounts of Pt, the nanowires sink into the silicon, forming an elliptical alloying regime. In the case of 4.3 nm Pt-film thickness, the resulting nanowires are almost completely sunken into the wafer surface. Alloying becomes a relevant contribution starting for Pt-film thicknesses of 2.2 nm and higher and then becomes dominant over the sheer nanowire formation. The nanowire formation follows the initial linear relationship between Pt-film thickness and nanowire width until it runs into self-limiting behavior at about 2.2 nm. 

**Figure 5:**
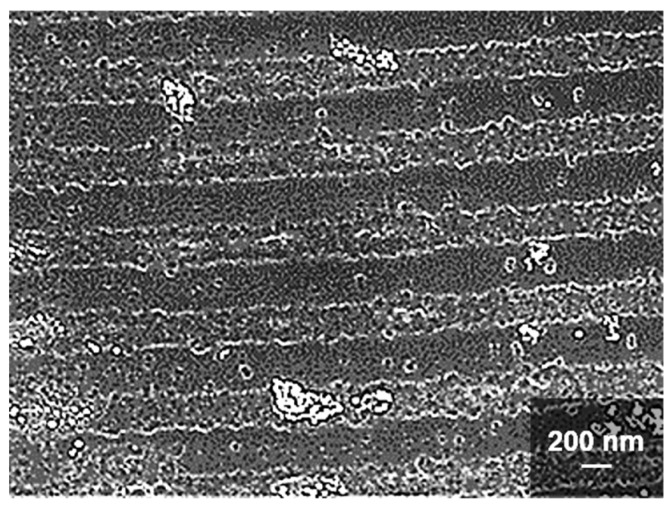
Etching image of a former Pt-LIPSS array. SEM image of a Pt-nanowire array obtained from LIPSS formation on Pt-films of 2.6 nm after etching with freshly prepared aqua regia and 1 M hydrofluoric acid.

Etching studies of the nanowires reveal the alloying of Pt from the thin top-layer and Si from the wafer. Due to the difference in acid resistance, platinum nanowires are completely removed, while silicon stays untouched. In Figure 5, a LIPSS pattern obtained from a 2.6 nm thin film is shown after treatment with freshly prepared aqua regia and hydrofluoric acid, revealing a negative template of the former Pt-nanowires. The ditch widths of 223 nm measured corresponds nicely to the nanowire widths before etching. 

**Figure 6:**
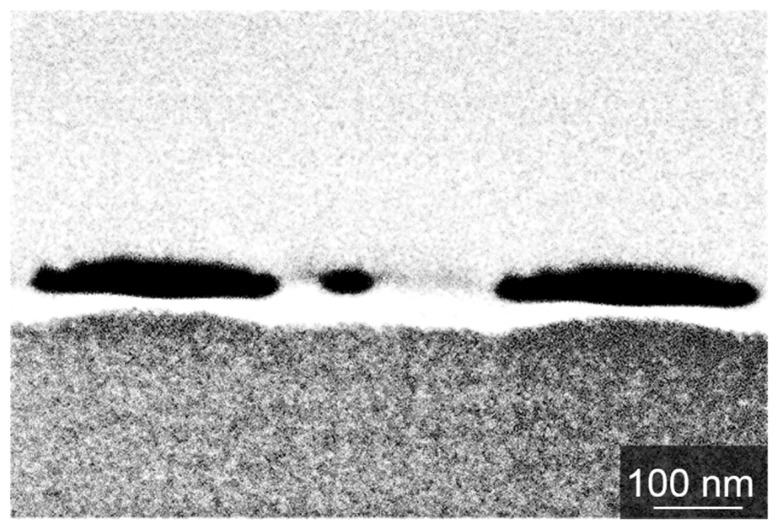
Dark field SEM image of a Pt-nanowire cross-section on silicon produced by focused ion beam slicing. Dark field SEM image of two Pt-nanowire cross-sections resulting from an initial 2.2 nm thick Pt-layer.

LIPSS-generation by nanosecond-pulsed lasers typically yields fused nanostructures of high quality. This is attributed to the fact that incident light interacts with the diffracted light near the surface of the sample, forming a periodic energy/heat pattern. The formation of the nanowires is mainly thermally driven, thus promoting the self-perfection of as-generated patterns by defect removal in the liquefied state [30]. In fact the propagating periodic heat pattern affects the silicon wafer surface as well (Figure 6), destroying the former crystalline surface and generating an amorphous layer. 

Considering a homogeneous illumination during LIPSS-generation, due to a standing wavefront of the laser, a periodic energy/heat intake into the sample occurs with a periodicity close to the wavelength of the laser light. Corresponding to the common theory of thermal LIPSS-formation, the material of the platinum layer accumulates in the local region of minimal energy. The amorphization of the silicon surface is maximal between the Pt-wires and minimal below. This observation strongly supports the theory of thermal LIPSS-formation.

**Figure 7:**
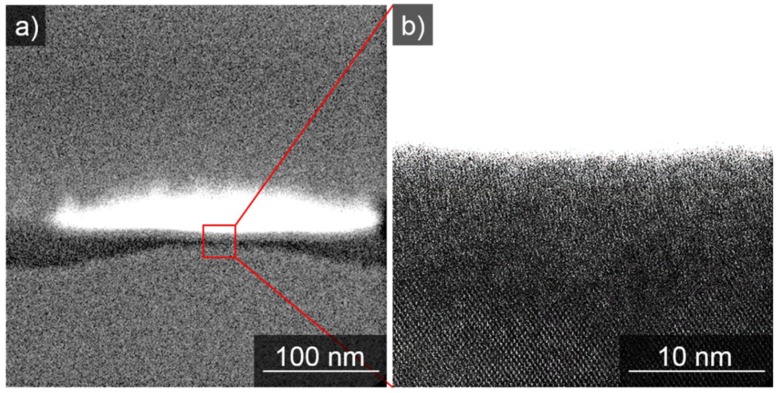
STEM images of a Pt-nanowire cross-section on silicon. STEM images of a Pt-nanowire cross-section resulting from an initial 2.2 nm thick Pt-layer. (**a**) The interface between the wafer and a Pt-wire. In (**b**) the area underneath the middle part Pt-wire is shown. Between platinum and the crystalline area of the silicon wafer, an amorphous region of roughly 10 nm thickness has formed.

The wafer crystallinity in the surface region disappears, forming an about 10 nm deep amorphous area, see Figure 7a, directly underneath the middle of the nanowire. In Figure 7b the amorphous depth of silicon increases as no protecting Pt-wire lies above. 

**Figure 8:**
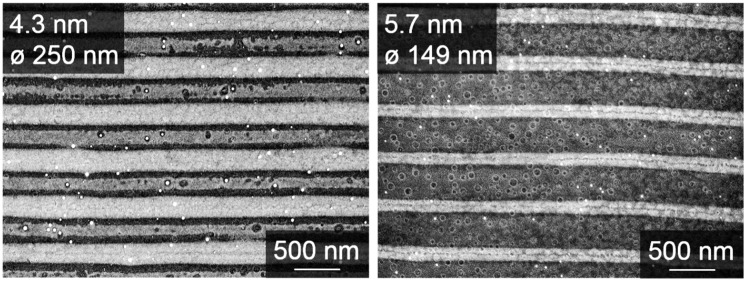
Critical Pt-film thickness for the occurrence of ablation effect. SEM images of Pt-nanowire arrays obtained from LIPSS formation on Pt-films of 4.3 nm (**left**) and 5.7 nm (**right**) thickness.

Pulsed energy absorption causes liquefaction and even evaporation of the metallic top layer and up to a certain depth of the silicon substrate too. [31]. The post-pulse extremely rapid cooling leads to the formation of continuous nanowires in the ‘cold’ areas of the LIPSS pattern. This is accompanied by non-equilibrium alloying of platinum and silicon. The less platinum available on the surface the thinner nanowires formed. Nanowires formed from 5.7 nm Pt-films feature average widths of 149 nm, whereas 4.3 nm Pt-films yield nanowires with average widths of 250 nm (Figure 8). It appears as if Pt thin films in excess of 5 nm thickness lose material upon laser irradiation under LIPSS-conditions. We suppose that increased photothermal coupling eventually leads to plasma formation accompanied by the evaporation of platinum. The effect is actually visible during LIPSS formation on Pt-films of increasing thickness.

## 4. Conclusions

Highly ordered Pt-nanowire arrays featuring an average periodicity of 538 nm are generated by nanosecond-pulsed laser-induced self-organization (LIPSS) of Pt thin films supported by silicon. The widths of Pt-nanowires are tunable in a range from 20 nm to 250 nm by adjusting the thickness of Pt-films in the range from 0.3 nm to 4.3 nm. A linear relation between the Pt-film thickness and the resulting width of the Pt-nanowires is found for Pt-nanowires generated from Pt-films in the range from 0.3 nm to 2.2 nm thickness. For Pt-films from 2.2 nm to 4.3 nm thickness, only a marginal increase in nanowire width is observed, thus indicating self-limiting behavior in this range. This is caused due to a superposition of surface tension of the platinum and the alloying enthalpy of platinum and silicon. LIPSS formation on Pt-films with 5.7 nm thickness results in even lower nanowire widths of about 150 nm which is only about 60 % of the nanowire widths obtained by LIPSS formation on Pt-films of 4.3 nm thickness (Pt-nanowire width 250 nm). Alloy formation between platinum and silicon is identified to be the reason for observed deviations from the initially linear relationship between Pt-film thickness and resulting nanowire width. LIPSS uses the top-layer excitation to structure the substrate-forming metastable alloy-like hybrid material nanowires due to the non-adiabatic rapid heating and cooling rates caused by nanosecond-pulsed laser excitation. The silicon substrate undergoes surface amorphization corresponding to the periodicity of the nanowires, strongly supporting the model of a thermal generation process of LIPSS. Another factor is the loss of material for nanowire formation from Pt-films exceeding a thickness of about 5 nm.

## Supplementary Materials

The Supplementary Materials are available online at https://www.mdpi.com/2079-4991/9/7/1031/s1.
