# Supplementary material for: Formation of Highly Ordered Platinum Nanowire Arrays on Silicon via Laser-Induced Self-Organization
Source: Nanomaterials (Basel). 2019 Jul 18;9(7):1031. doi: 10.3390/nano9071031 (PMC6669604; doi:10.3390/nano9071031)
Supplement: Supplementary file 1 [file nanomaterials-09-01031-s001.pdf]

Supplementary

Formation of Highly Ordered Platinum Nanowire Arrays on Silicon via Laser-Induced Self-Organization

Following image shows the scheme of the used laser setup.

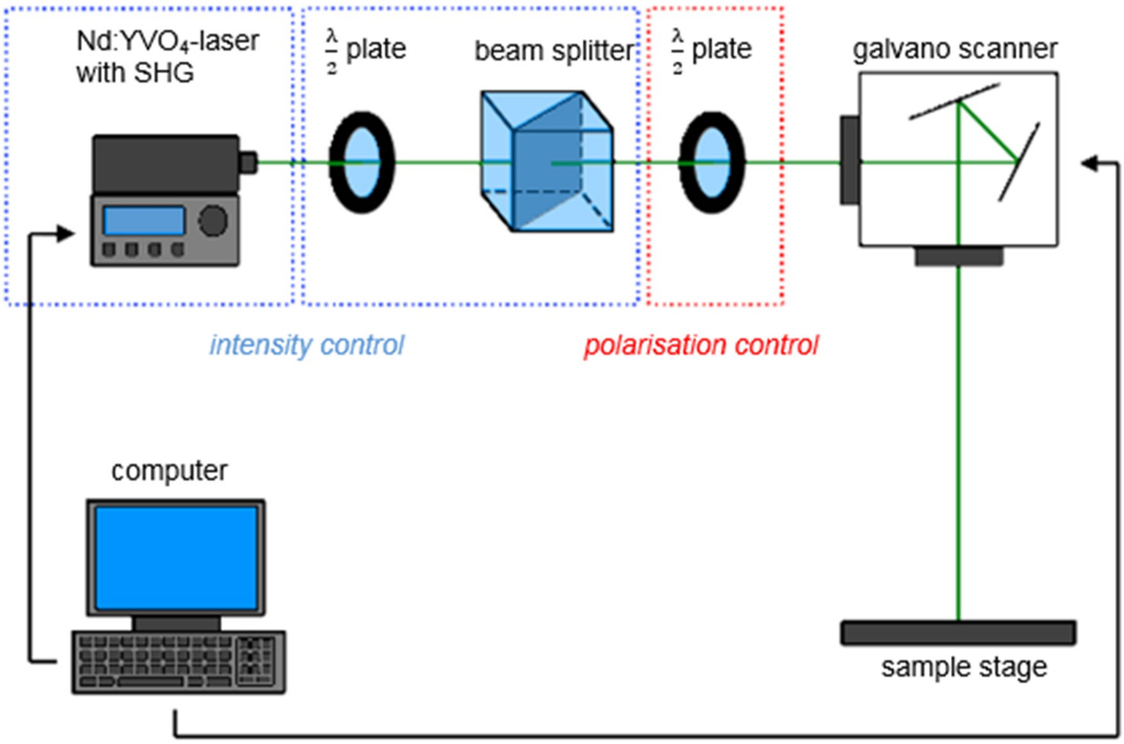

Supplementary Figure S1. Scheme of the used laser setup.
A Nd:YVO_4-laser with second harmonic generation-crystal illuminates the sample through a $\lambda/2$ plate, followed by a beam splitter regulating the intensity of the beam. The polarisation of the laser beam is be controlled via a second $\lambda/2$ plate. A Galvano scanner is used to move the laser spot over the sample.
